# Supplementary material for: Design, Synthesis and Biological Evaluation of Novel PEG-Rakicidin B1 Hybrid as Clostridium difficile (CD) Targeted Anti-Bacterial Agent
Source: Molecules. 2023 Aug 21;28(16):6152. doi: 10.3390/molecules28166152 (PMC10457740; doi:10.3390/molecules28166152)
Supplement: Supplementary file 1 [file molecules-28-06152-s001.zip › molecules-2557999-supplementary.pdf]

Supplementary Material

Table of Contents

Section	Title	Page
**Figure S1-S11**	Characterization of **compound**	2-7
**Table S1**	Anticancer activity by Hypoxia and Normoxia against HCT-8, PANC-1, Caco-2 cells.	7
**Table S2**	The NMR Spectroscopic Data of FIMP2	7-8

Characterization of Z-01.

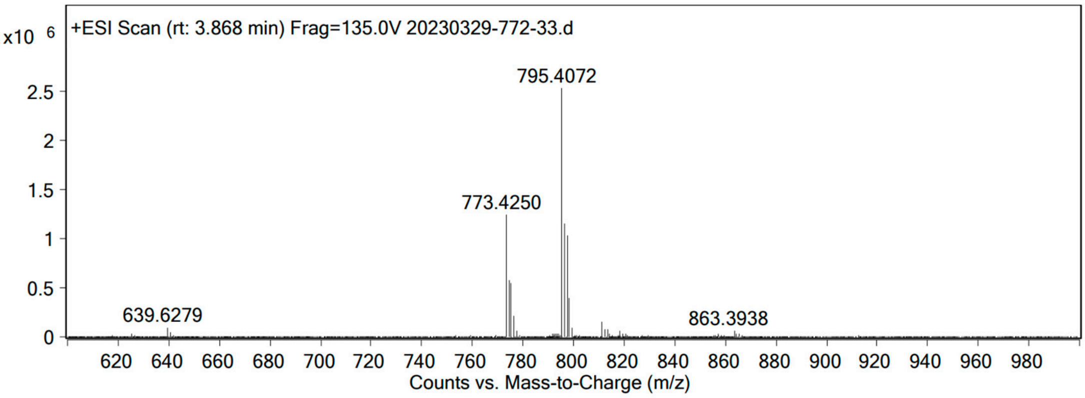

Figure S1. HRMS spectrum of **Z-01.**

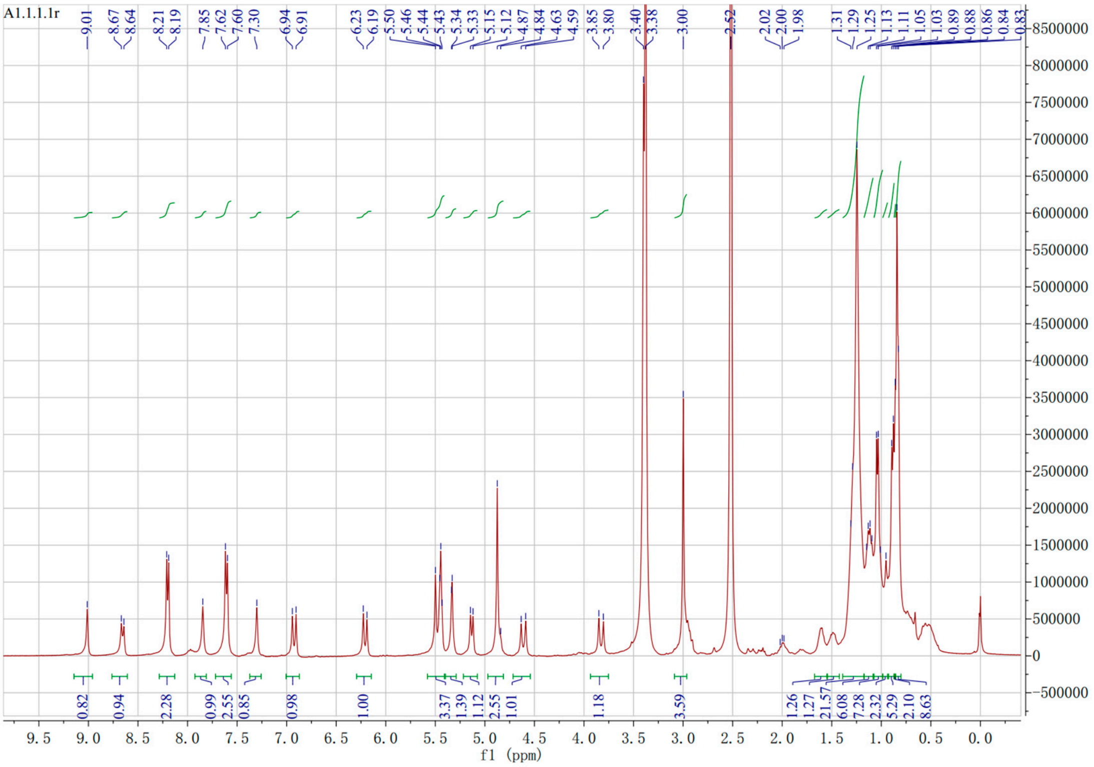

Figure S2. ^{1}H NMR spectrum of **Z-01** in DMSO-*d6*.

Characterization of Z-02.

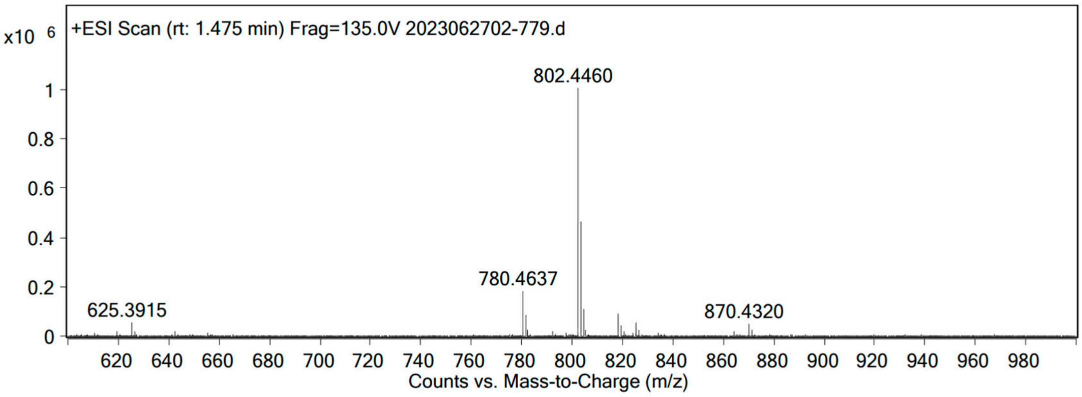

Figure S3. HRMS spectrum of **Z-02.**

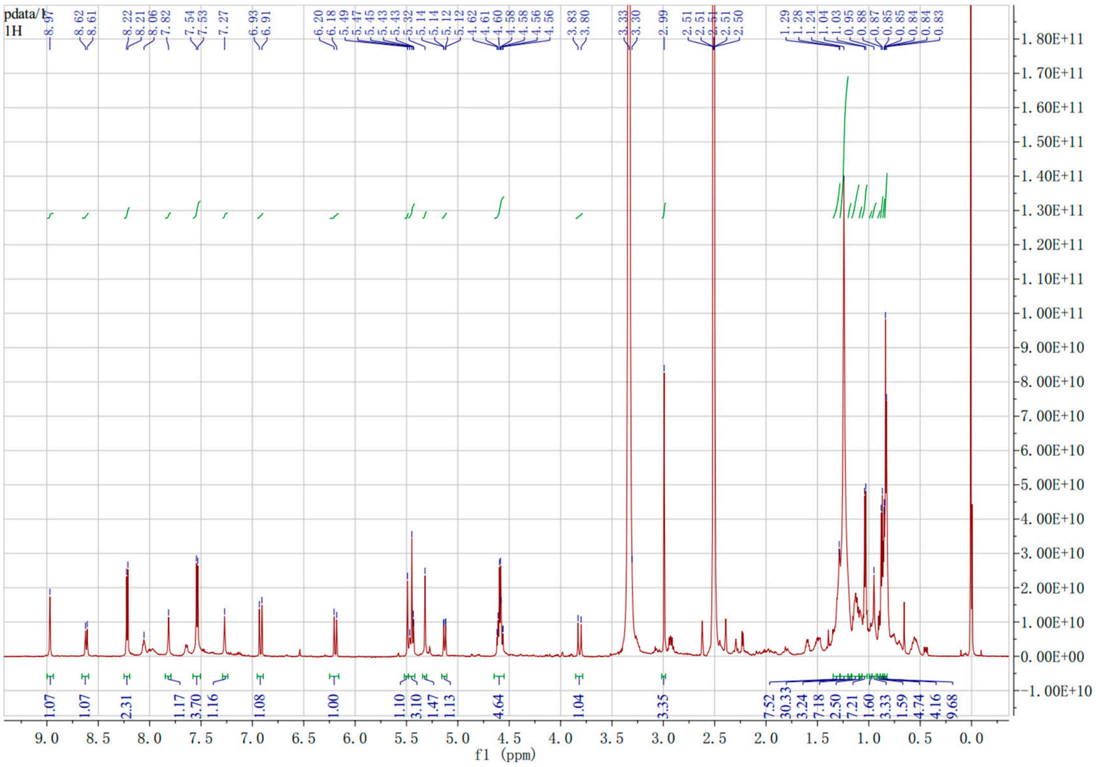

Figure S4. ^{1}H NMR spectrum of **Z-02** in DMSO-*d6*.

Characterization of FIMP2.

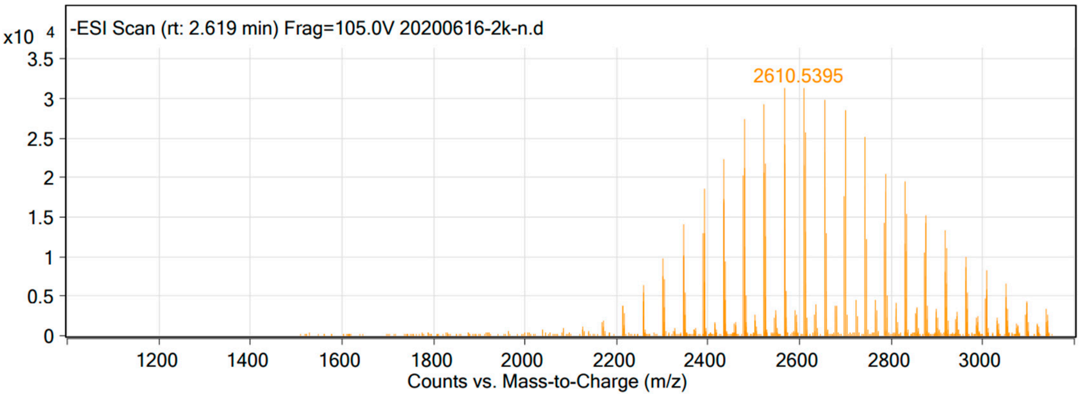

Figure S5. HRMS spectrum of **FIMP2.**

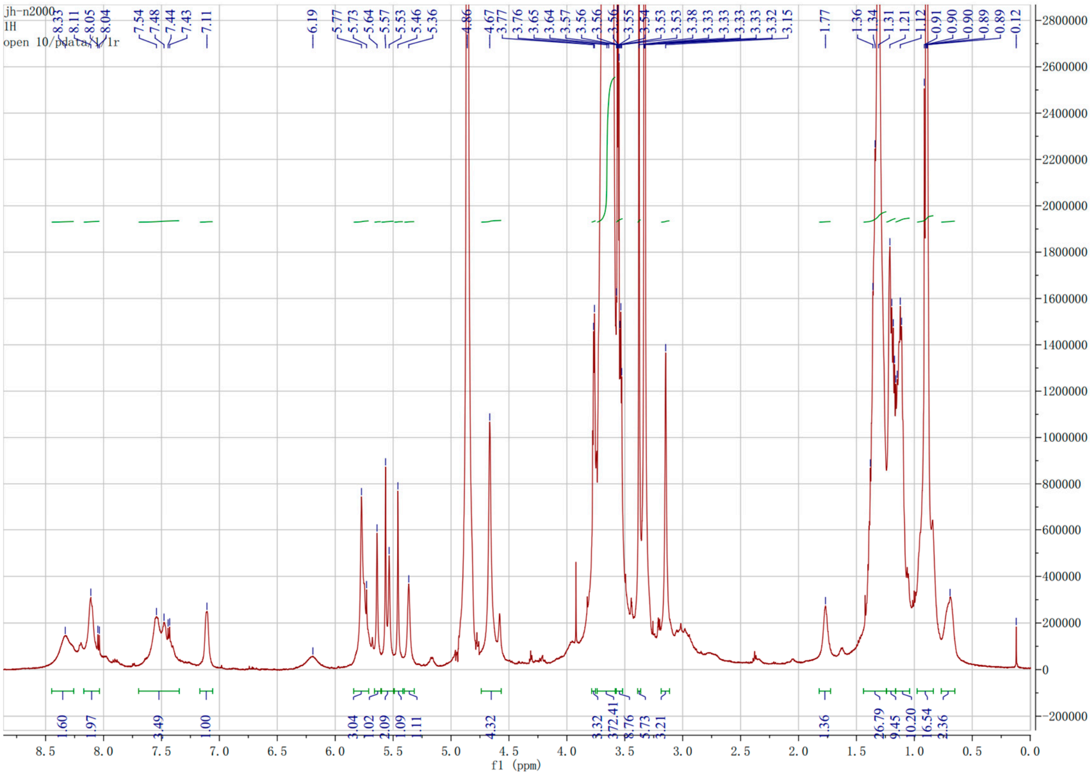

Figure S6. ^{1}H NMR spectrum of **FIMP2** in DMSO-d_6.

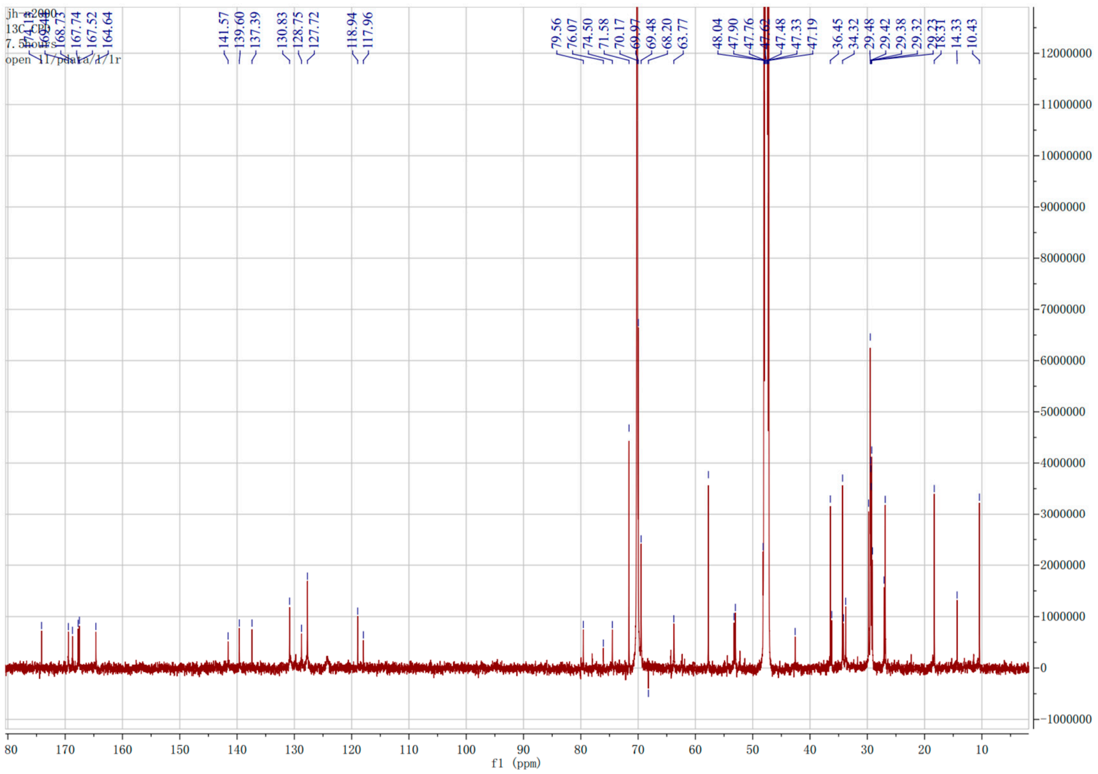

Figure S7. ^{13}C NMR spectrum of **FIMP2** in DMSO-d_6.

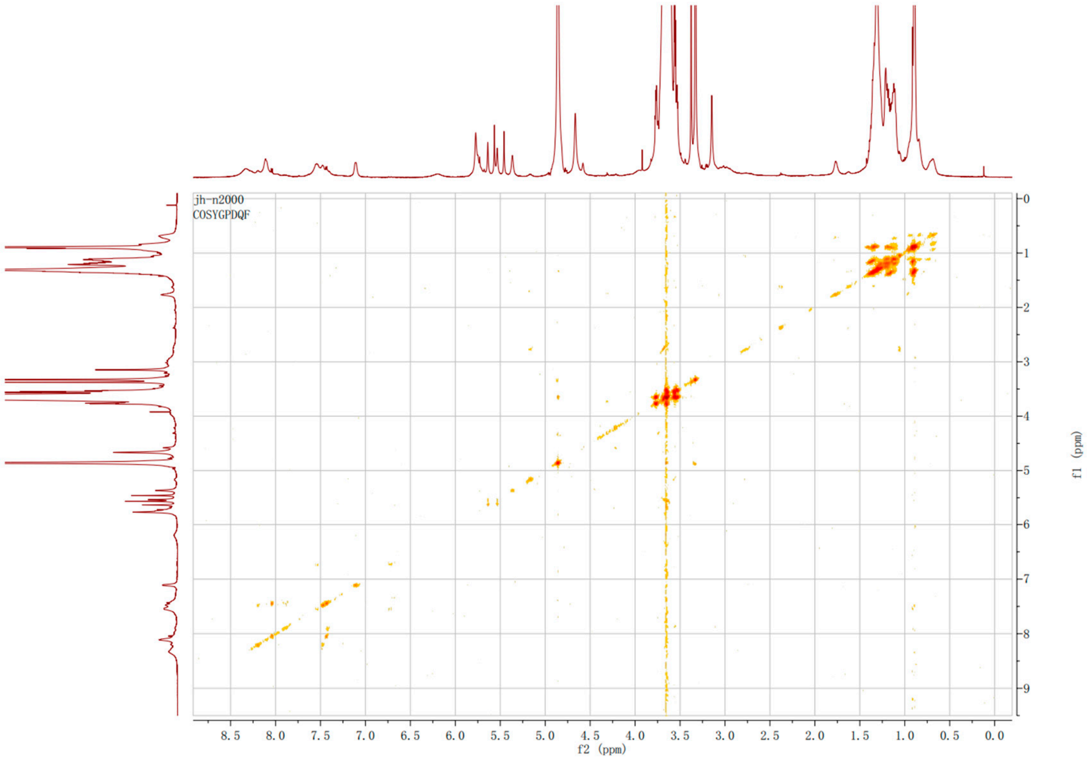

Figure S8. COSY spectrum of **FIMP2.**

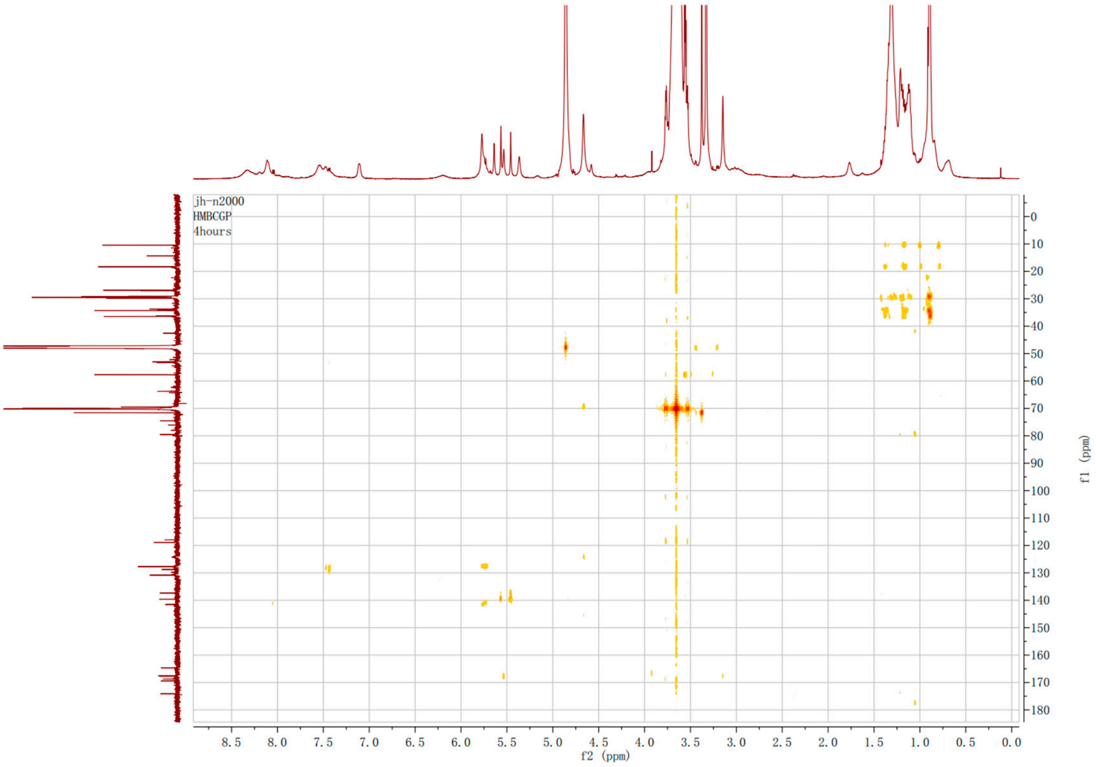

Figure S9. HMBC spectrum of **FIMP2.**

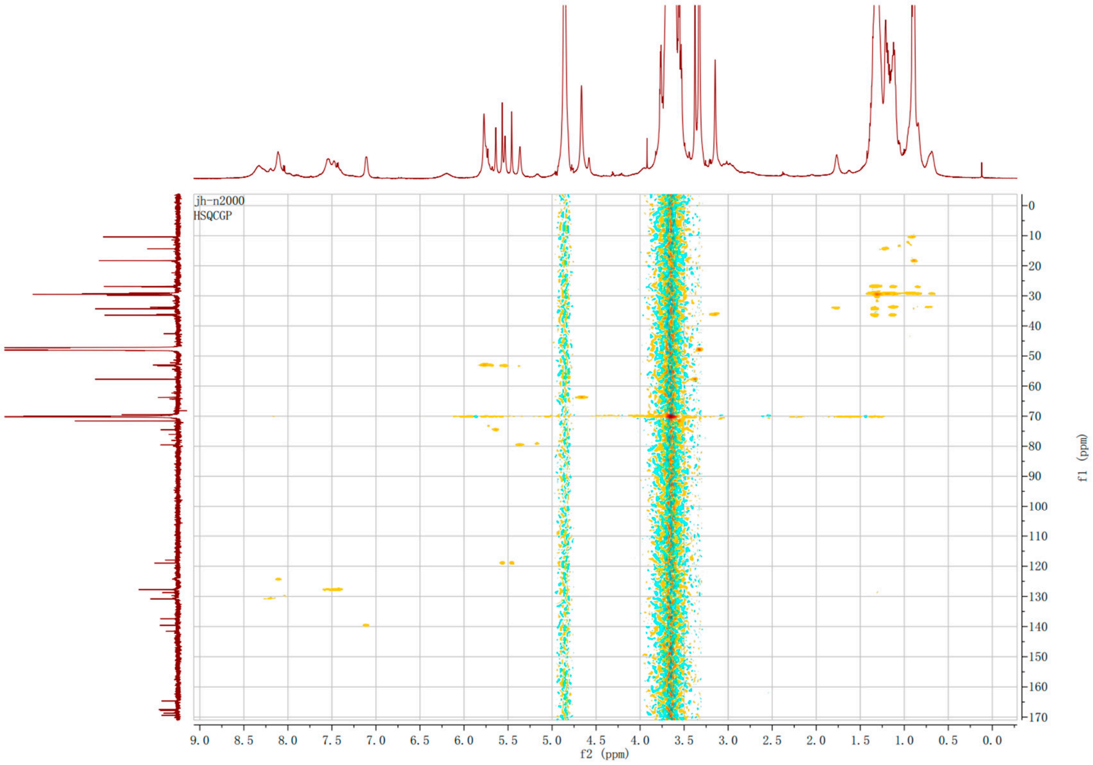

Figure S10. HSQC spectrum of **FIMP2.**

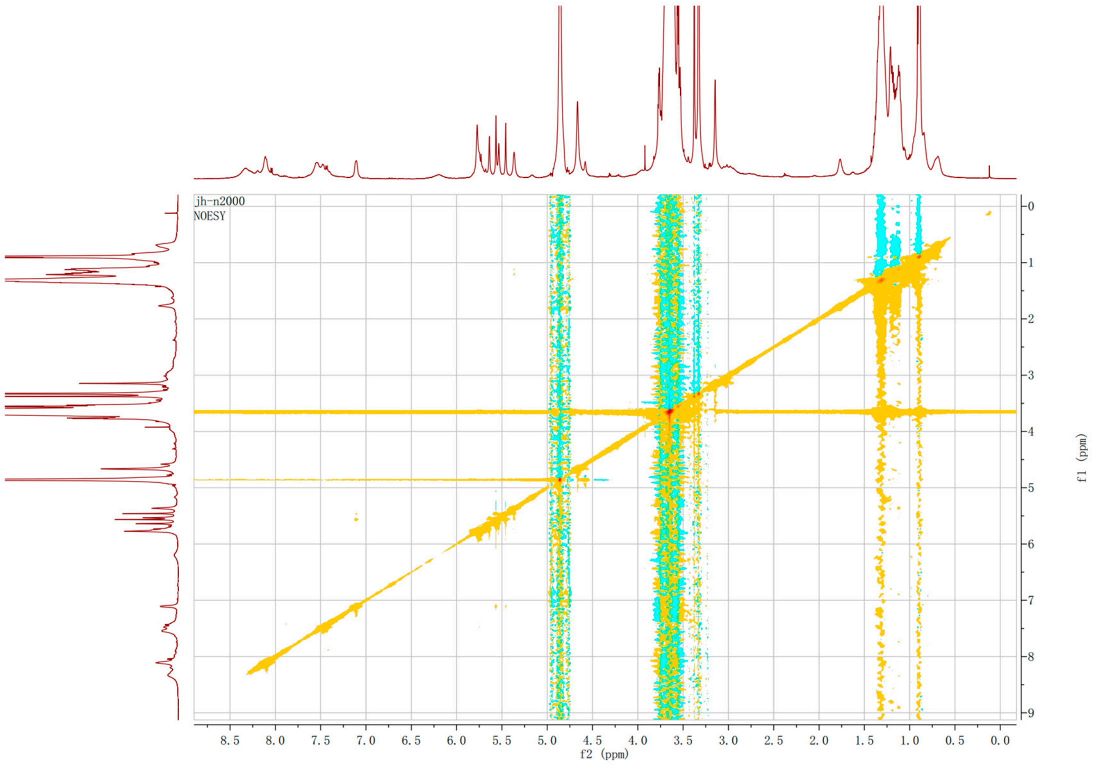

Figure S11. NOESY spectrum of **FIMP2.**

Table S1. Anticancer activity by Hypoxia and Normoxia against HCT-8, PANC-1, Caco-2 cells.

conditions	compound	IC_{50}(μM)		
		HCT-8	PANC-1	Caco-2
Normoxia	B1	0.519	0.815	0.586
	FIMP2	47.641	40.79	39.505
Hypoxia	B1	0.43	0.486	0.457
	FIMP2	34.395	36.205	30.482

Table S2 NMR Spectroscopic Data (600 MHz, DMSO-*d6*) for **FIMP2** in DMSO-*d6.*

Position	δ_C, mult.	δ_H
1	167.7, qC	-
2	53.3, CH	5.53
3	74.5, CH	5.64
4	174.1, qC	-
5	167.5, qC	-
6	53.0, CH_2	5.77
7	36.2, CH_3	3.15, s
8	164.6, qC	-
9	118.0, CH	

10	139.6, CH	7.11
11	137.4, qC	-
12	118.9, CH_2	5.57, s
		5.46, s
13	169.4, qC	-
14	57.8, CH	3.38
15	79.6, CH	5.36
16	34.3, CH	1.36, m
17	33.8, CH_2	1.11, m
		0.69, m
18	27.1, CH_2	1.12, m
		0.84, m
19	29.4, CH_2	1.32, m
20	29.4, CH_2	1.32, m
21	29.4, CH_2	1.32, m
22	29.4, CH_2	1.32, m
23	29.4, CH_2	1.32, m
24	29.4, CH_2	1.32, m
25	29.4, CH_2	1.32, m
26	26.9, CH_2	1.34, m
27	36.5, CH_2	1.35, m
		1.13, m
28	34.2, CH	1.77, m
29	29.1, CH_2	0.93, m
30	10.4, CH_3	0.90
31	14.3, CH_3	1.21
32	13.2, CH_3	1.12
33	18.3, CH_3	0.89, d (2.4)
1′	168.7	
2′	128.8	
3′	130.8	8.33, m
4′	127.7	7.50, m
5′	141.6	
6′	127.7	7.50, m
7′	130.8	8.33, m
8′	63.8	4.67, s
9′	124.3	
10′	129.8	8.11, s
